# Comparison of Medical Imaging Quality Related to Embalming Solutions in Canine Cadavers

**DOI:** 10.3390/vetsci12020188

**Published:** 2025-02-19

**Authors:** Ahsa Oh, Sung-Min Nam, Sang-Soep Nahm, Ki-Dong Eom, Woosuk Kim

**Affiliations:** 1 Department of Veterinary Medical Imaging, College of Veterinary Medicine, Konkuk University, 120 Neungdong-ro, Gwangjin-gu, Seoul 05030, Republic of Korea; iamahssa@naver.com (A.O.); eomkd@konkuk.ac.kr (K.-D.E.); 2 Department of Anatomy, School of Medicine and Jesaeng-Euise Clinical Anatomy Center, Wonkwang University, Iksan 54538, Republic of Korea; namvet1@wku.ac.kr; 3 Department of Anatomy, College of Veterinary Medicine, Konkuk University, 120 Neuugdonr-ro, Gwangjin-gu, Seoul 05030, Republic of Korea; ssnahm@konkuk.ac.kr; 4 College of Veterinary Medicine and Veterinary Science Research Institute, Konkuk University, 120 Neungdong-ro, Gwangjin-gu, Seoul 05030, Republic of Korea

**Keywords:** medical imaging quality, canine cadaver, formalin solution, Thiel solution, saturated salt solution

## Abstract

(1) Background: This study evaluated the quality of medical imaging acquired using radiography, ultrasonography, and computed tomography (CT) in canine cadavers prepared with formalin solution, Thiel solution (TS), and saturated salt solution (SS). Additionally, the duration whereby image quality could be properly reproduced was investigated. (2) Methods: Nine dog cadavers, divided into three groups per embalming solution, were imaged pre- and post-embalming at 6 and 12 weeks using three different imaging modalities. Image quality was qualitatively scored based on human medicine guidelines criteria. The score was assigned as follows: “0 = not invisible, 1 = poorly invisible, 2 = adequately reproduced, 3 = very well reproduced”. (3) Results: On plain radiography, most scores were > 1.34, although the quality of all embalming solutions in thoracic and abdominal images decreased over time. Using ultrasound to image the liver, spleen, and kidney, the SS group was evaluated as poorly visible and scored < 0.82 at 6 and 12 weeks. CT images showed a tendency to degrade over time, but all solution groups had properly implemented images and scored > 1.62. (4) Conclusions: Regarding image quality and ease of application, TS is considered the most suitable embalming solution for medical imaging acquisition and is usable over time in all imaging modalities.

## Simple Summary

To the best of our knowledge, this is the first study to compare images obtained over time to assess the long-term usefulness of embalmed canine cadavers in veterinary medical imaging education. The three embalming solutions used in this study have different weaknesses in terms of the degree of degradation of imaging quality and ease of examination of imaging modalities, including radiography, ultrasound, and CT. In conclusion, TS is the most appropriate embalming solution for all the imaging techniques used in veterinary medical imaging education.

## 1. Introduction

Canine cadavers are utilized in teaching, training, and veterinary medical education, playing a crucial role in learning and understanding normal anatomical structures in both basic and clinical subjects [1,2,3,4,5,6,7]. However, the use of animal models in veterinary education has become increasingly challenging owing to growing awareness of animal welfare and the strong push for applying the 3R (Replacement, Reduction, and Refinement) principle [8]. This has led to a greater emphasis on the long-term preservation and multiple uses of canine cadavers, while minimizing the number of animals euthanized in the educational process.

Radiological imaging provides a three-dimensional perspective that enhances the understanding of normal anatomy and aids in learning clinical subjects [9,10]. Some imaging modalities, such as computed tomography (CT) and magnetic resonance imaging (MRI), require anesthesia for patient examination, but repeatedly anesthetizing a limited number of experimental dogs is not feasible. Therefore, using canine cadavers to study these imaging techniques, which require anesthesia, is a necessary consideration. Additionally, cadavers can be utilized for training in invasive procedures that require ultrasound guidance [5].

Embalming is the process of injecting chemicals into the body to prevent tissue de-composition [11]. Various embalming methods have been developed, incorporating disinfectants, preservatives, and fixatives [11,12,13,14,15,16]. Formalin solution (FS) is one of the most common embalming agents owing to its ability to cross-link tissue proteins, stop self-degradation, and prevent microbial infection, thereby preserving anatomical structures for extended periods [11,17]. As a result, formalin-embalmed cadavers are widely used in medical education [9,18,19].

However, the high concentration of formalin in traditional embalming solutions can lead to several drawbacks, including tissue edema, which degrades image quality [20]. Formalin also irritates the mucous membranes, such as the eyes and respiratory system, and is considered a carcinogen with prolonged exposure, posing a health risk to users [13,21]. Additionally, tissue hardening caused by formalin makes it difficult to position cadavers accurately [18,22]. For these reasons, the use of formalin is highly restricted. In the United States, Occupational Safety and Health Administration (OSHA) regulations outlined in 29 CFR 1910.1048 specify permissible exposure limits (PEL), stating that exposure must not exceed a time-weighted average of 0.75 ppm over an 8 h period. Additionally, a short-term exposure limit (STEL) is set, prohibiting exposure levels from exceeding 2 ppm over a 15 min period. Given these restrictions, the use of formalin as an embalming solution for preparing cadavers in veterinary education should be reduced compared to its traditional usage. To address these issues, several alternative embalming solutions have been proposed [2,6,13,14,17,23,24]. Thiel solution (TS) and saturated salt solution (SS) have shown promise as alternatives in recent studies [1,3,6,11,14,15,18,22,25,26]. These soft tissue preservative solutions have demonstrated effectiveness in human cadaver studies in Europe and Japan and have since been applied to canine cadavers for veterinary education [1,3,7,14,15,16,18,22,25,26].

This study aims to evaluate the effectiveness of alternative embalming solutions in veterinary imaging medicine education. Specifically, it compares the radiological quality of images from TS and SS cadavers with those from FS cadavers. The study also examines the cadavers over time to assess the quality of imaging and their long-term usability in educational settings.

## 2. Materials and Methods

### 2.1. Animals

Nine adult beagles (Raon Bio Inc., Yongin, Republic of Korea) were enrolled in this study and divided into three groups. The three groups included the formalin solution embalming group (FS), the saturated salt solution embalming group (SS) and the Thiel’s solution embalming group (TS), with each group consisting of three animals. All dogs were intact with no prior history of disease and had a mean weight of 11.9 kg (range, 8.2–14.3 kg). This study was approved by the Institutional Animal Care and Use Committee of the Konkuk University (approval no. KU 18132).

### 2.2. Embalming Solutions and Protocol

Embalming solutions were prepared to produce canine cadavers. The three embalming solutions (Table 1) were based on the combination used in a previous study [14]. Briefly, the FS contained formalin (5%, Junsei Chemical Co., Tokyo, Japan), ethanol (Duksan Pure Chemical Co., Ltd., Ansan, Republic of Korea), phenol (Daejung Chemicals & Metals Co., Siheung, Republic of Korea), glycerol (Junsei Chemical Co.), and water. TS contained formalin (2.12%, Junsei Chemical Co.), chloromethyl phenol (Acors Organics Co., Gael, Belgium), propylene glycol (Duksan Pure Chemical Co., Ltd.), ammonium nitrate (Samcheon Pure Chemical Co., Ltd. Seoul, Republic of Korea), boric acid (Sigma-Aldrich, St. Louis, MO, USA), potassium nitrate (Duksan Pure Chemical Co., Ltd.), morpholine (Daejung Chemicals & Metals Co.), and ethanol (Duksan Pure Chemical Co., Ltd.). The SS contained sodium chloride (4 kg/4.52 L of water, Junsei Chemical Co.), formalin (2.68%, Junsei Chemical Co.), isopropyl alcohol (Duksan Pure Chemical Co., Ltd.), phenol (Daejung Chemicals & Metals Co.), and glycerol (Junsei Chemical Co.).

Following sedation with 0.03 mg/kg medetomidine (Domitor^®^, Pfizer Animal Health, Walton Oaks, UK), the dogs in each group were anesthetized using a gas mixture comprising 2.5% isoflurane (Baxter Healthcare, Deerfield, IL, USA), 33% oxygen, and 67% nitrous oxide. Then, the dogs in each group were euthanized by exsanguination, with blood extracted from the common carotid artery using a 50 mL syringe. An additional 1.5 L of embalming solution appropriate for each group (FS, SS, and TS) was perfused using a peristaltic pump and 50 mL was injected into the thoracic and abdominal cavities. Eight hours post-embalming, the cadavers were sealed in plastic bags and stored in a refrigerator at 2–4 °C. 

### 2.3. Imaging Protocol and Assessment Criteria

Image acquisition was conducted four times, and the time points were defined as follows: pre, before euthanasia; 0 w, the day after embalming; 6 w, 6 weeks after embalming; 12 w, 12 weeks after embalming. All cadavers underwent three different imaging modalities: radiography, ultrasonography, and CT. Assessment criteria and a qualitative grading system for each imaging modality were adapted from the European Guidelines on Quality Criteria for Diagnostic Radiographic Images, the American Institute of Ultrasound in Medicine (AIUM) Practice Guideline for ultrasound, and European Guidelines on Quality Criteria for Computed Tomography of Human Medicine [27,28]. Images were saved as digital imaging and communication in medicine files and loaded into post-processing software (Radiant^TM^ version 3.4.1, Medixant, Poznan, Poland). Three radiologists performed qualitative scoring independently. To avoid bias, the readers were blinded to the kind of embalming solution and examination duration.

### 2.4. Plain Radiographic Examination of Thoracic and Abdominal Cavity

Plain Radiographs of thorax and abdomen were performed with digital radiography units (Titan 2000^®^ V., Comed Medical system, Seoul, Republic of Korea). Radiographic images of the right lateral and ventrodorsal (VD) projections were obtained. The kilovoltage peak and milliamperes were set according to the thoracic and abdominal thickness of the dogs.

Two readers qualitatively graded the images from 0 to 3 as follows: 0, not visible; 1, poorly visible; 2, adequately reproduced; 3, excellent reproduced. To facilitate the comparison, the mean score was calculated by dividing the total original score by the number of criteria used.

**Figure 1:**
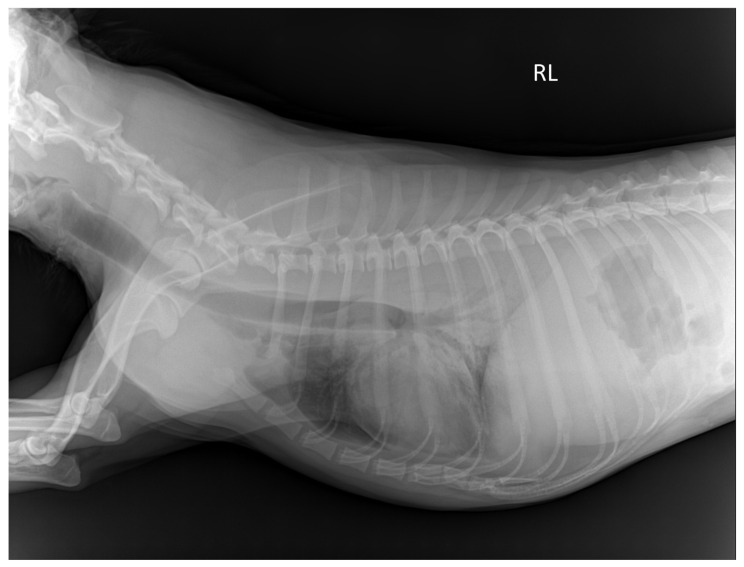
Radiographic image of the thorax of a TS cadaver 12 weeks post-embalming. RL: Right lateral recumbency. The mean score for thorax is 1.58; trachea and proximal bronchi is 2; descending aorta is 0.5; endplates of thoracic vertebrae is 3; cardiac border is 2; costophrenic angle is 1; and lung parenchyma is 1. The scores were assigned as follows; 0 = not visible, 1 = poorly visible, 2 = adequately reproduced, 3 = excellent reproduced.

On the thoracic image, the visibility of the trachea and proximal bronchi, descending aorta border, upper and lower endplates of the thoracic vertebrae, cardiac border, costophrenic angles, and lung parenchyma were rated (Figure 1). Radiologists assessed the visibility of the spinous process of the lumbar spine, outline of the cranial and caudal poles of the left kidney, and outline of the urinary bladder in the abdominal area.

### 2.5. Ultrasonographic Examination of Abdominal Organ

Ultrasonography of the abdominal organs, including the liver, spleen, and kidney, was performed by a single radiologist using a linear transducer (3.4–13 MHz, UST-5415, Hitachi-Aloca Medical, Ltd., Tokyo, Japan) and ultrasonographic machine (Prosound F75^®^; Hitachi-Aloca Medical, Ltd.). The representative images were recorded and reviewed by another radiologist.

**Figure 2:**
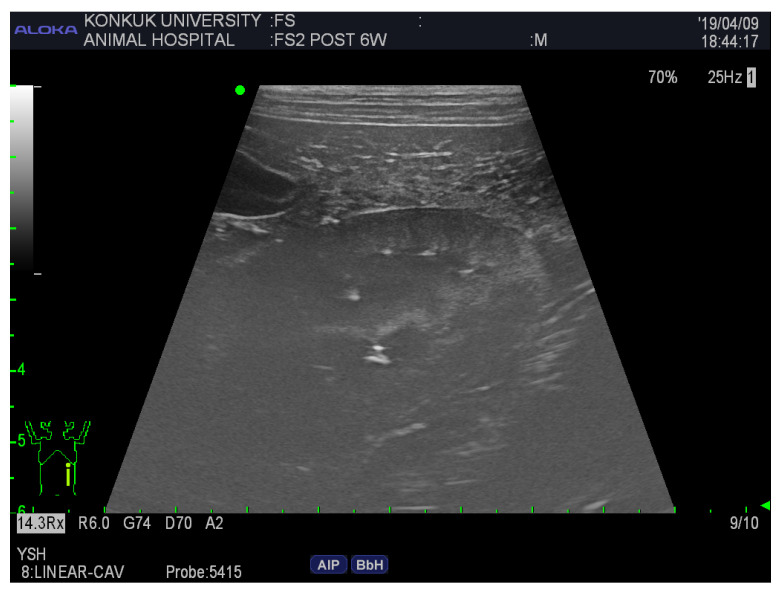
Ultrasonographic image of the kidney of an FS cadaver 6 weeks after embalming. The mean score was 2. Both poles at long axis = 1, smooth margination = 3, corticomedullary junction = 2. The scores were assigned as follows; 0 = not visible, 1 = poorly visible, 2 = adequately reproduced, 3 = excellent reproduced.

The visibility of the following abdominal organs was scored as follows: liver, distension of the gallbladder, coarse echotexture; spleen, smooth margination, fine echotexture; kidney, both poles visible along the long axis, smooth margination, corticomedullary junction (Figure 2); and the relative echogenicity of the spleen, liver, and kidney.

### 2.6. Computed Tomographic Examination of Whole Body

Whole-body multi-detector scanning was performed by a radiologist using a 4-channel helical CT scanner (LightSpeed^®^, GE Healthcare, Milwaukee, WI, USA). The cadavers were scanned craniocaudally for ventrodorsal/dorsoventral (VD/DV) positioning. CT acquisitions were performed using the following parameters: 200 mA, 120 kVp, 1.25 mm helical thickness. Before euthanasia, a CT scan was performed under general anesthesia with controlled respiratory conditions.

**Figure 3:**
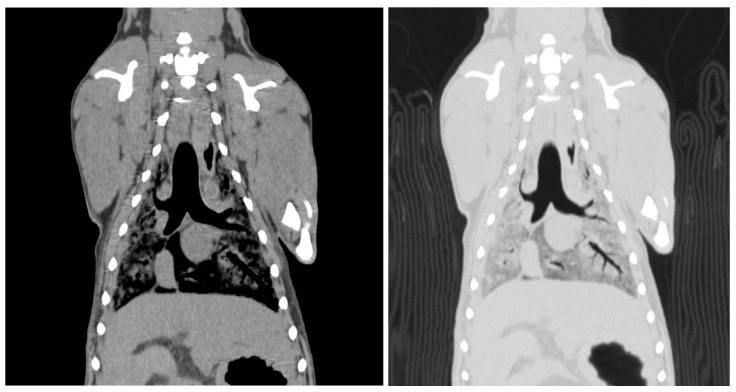
Computed tomographic image of the thorax of an SS cadaver 12 weeks after embalming. These images were reconstructed with soft tissue and a lung window. The mean score is 2.50. Lung parenchyma = 1, heart = 3, thoracic aorta = 3, cranial vena cava = 2.5, thoracic vertebra = 3. The scores were assigned as follows; 0 = not visible, 1 = poorly visible, 2 = adequately reproduced, 3 = excellent reproduced.

Two readers assessed the image quality of the CT scans. The visibility of the skull and lateral ventricle of the cerebrum in the head region was rated. The visibility of the thoracic structures, including the lung parenchyma, heart, aorta, cranial vena cava, and vertebrae, was scored (Figure 3). The visibility of the aorta, caudal vena cava, liver, kidney, spleen, and vertebrae was assessed.

### 2.7. Statistical Methods

All statistical analyses were performed using a commercial statistical software (SPSS Statistics version 25.0; IBM Corp., Armonk, NY, USA). Interclass correlation coefficient (ICC) tests were conducted to assess the level of consensus among radiologists. The visibility score of plain radiographs was calculated as the mean of two average ratings for the thorax and abdomen, whereas the mean of three average ratings was used for CT scans of the head, thorax, and abdomen. Repeated-measures analysis of variance (ANOVA) and the Bonferroni method were performed to determine whether the embalming solutions had a significant effect on image quality scores over time. Otherwise, the same analysis was used to calculate the difference compared to the pre-score. In all analyses, a *p*-value < 0.05 was considered significant, and all results were presented as mean scores.

## 3. Results

### 3.1. Subsection

The ICC for the image quality of plain radiography and CT images showed significant agreement (*p* < 0.001). The ICC values for each imaging modality were as follows: plain radiography ICC = 0.893 and CT ICC = 0.978; Table 2).

### 3.2. Visibility of Plain Radiographic Examination

**Figure 4:**
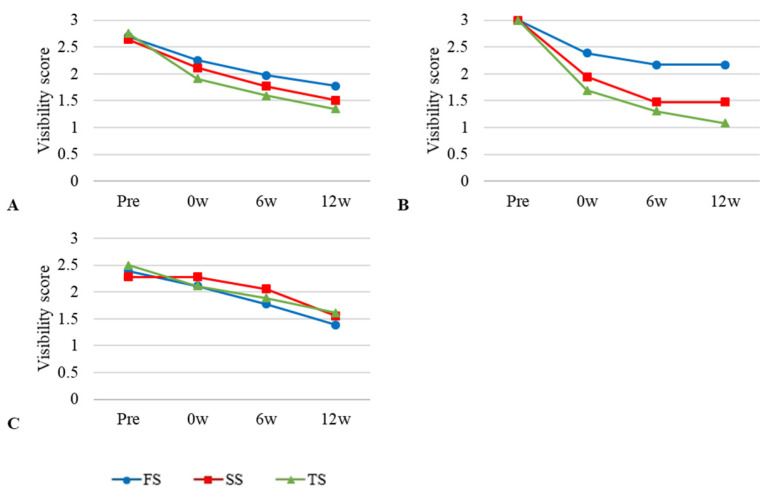
General and regional visibility scores of radiographic images at four time points using three different embalming solutions. (**A**) = Entire body; (**B**) = thorax; (**C**) = abdomen. The scores were assigned as follows; 0 = not visible, 1 = poorly visible, 2 = adequately reproduced, 3 = excellent reproduced.

The mean values and standard deviations of the plain radiograph visibility scores at the four time points are shown in Table 3. With all the embalming solutions, the quality of the images tended to deteriorate over time (Figure 4), although there was no statistically significant interaction between time and the embalming solution.

Repeated-measures ANOVA indicated that there was no significant difference in image quality among the embalming solution groups (*p* > 0.05). In addition, there was no statistical significance in the comparisons between the groups at any point in time (F_1,2_ = 0.726, F_2,3_ = 1.000, and F_3,1_ = 1.000).

### 3.3. Visibility of Ultrasonographic Examination

**Figure 5:**
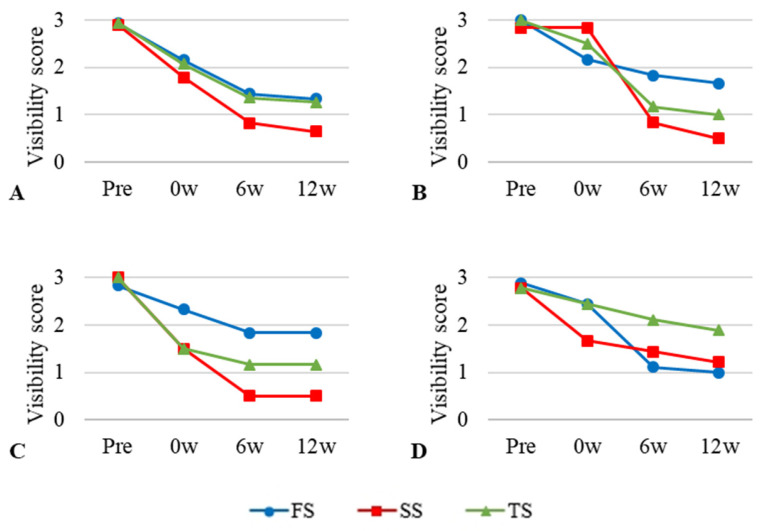
General and regional visibility scores of ultrasonographic images at four time points using three different embalming solutions. (**A**) = entire body; (**B**) = liver; (**C**) = spleen; (**D**) = kidney. The scores were assigned as follows; 0 = not visible, 1 = poorly visible, 2 = adequately reproduced, 3 = excellent reproduced.

The mean value and standard deviation of the visibility scores of the ultrasound images at the four time points are given in Table 4. An identical pattern of reduced ultrasound image quality was observed over time in all embalming solutions, with the difference being the largest among the three imaging modalities (Figure 5). The relative echogenicity of SS was scored less than one point at 6 weeks and 12 weeks. Liver images showed higher scores for TS and SS at 0 weeks, whereas FS scores were the highest after 6 weeks. In the spleen images, FS received the highest score at all time points. The image quality score of the kidney was reduced by more than one point compared to the pre-score for SS at 0 week. At 6 and 12 weeks, the kidney score was the lowest in the FS group. Overall, the visibility of the SS was judged to be poor, scoring less than one point from 6 weeks.

Repeated-measures ANOVA was performed to assess the effectiveness of the embalming solution and time, resulting in no significant effect over time (*p* = 0.098).

### 3.4. Visibility of Computed Tomographic Examination

**Figure 6:**
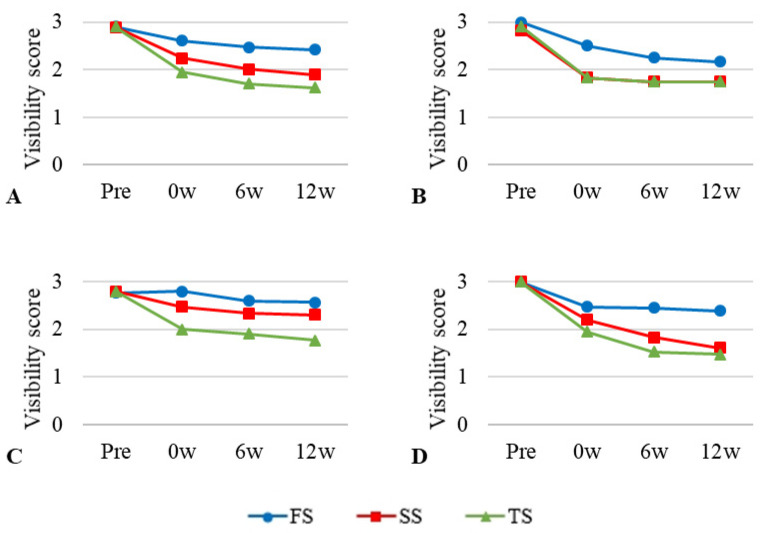
General and regional visibility scores of computed tomographic images at four time points using three different embalming solutions. (**A**) = entire body; (**B**) = head; (**C**) = thorax; (**D**) = abdomen. The scores were assigned as follows; 0 = not visible, 1 = poorly visible, 2 = adequately reproduced, 3 = excellent reproduced.

The mean values and standard deviations of the visibility scores of the CT images captured at the four time points are listed in Table 5. A decreasing tendency in image quality was observed over time in all the embalming-solution groups (Figure 6).

Repeated-measures ANOVA was performed to assess the effects of the embalming solution over time. Statistically significant differences in image quality between FS and TS were noted at weeks 0 (F_1,2_ = 8.184, *p* < 0.01), 6 (F_1,2_ = 2.571, *p* < 0.01), and 12 (F_1,2_ = 5.354, *p* < 0.01). There were no statistically significant differences between the SS and other groups.

The visibility scores of the CT images at all four time points and three area scores for the three different embalming solutions are shown in Figure 6. Over time, the CT visibility of all three embalming solutions steadily decreased (Graph A). Repeated ANOVA showed that the FS and TS groups differed significantly at post-embalming time points because of the embalming solutions (*p* < 0.01). The TS and SS scores in the head region were identical, and there was a rapid deterioration in the quality of the head images after embalming (Graph B). Overall, the image quality was the highest in the FS group. The image quality score for CT images at last examination was 1.62 for TS and 1.89 for SS (Graph A).

## 4. Discussion

This study demonstrated the usefulness of soft tissue preservative embalming solutions in veterinary medical imaging education. Although most veterinary medical schools still use conventional embalming solutions, several attempts have been made to use different teaching and training methods [7,16,22,29]. The results of this study indicated that TS is useful in veterinary imaging medicine education using cadavers. In particular, the results emphasize the utility of TS cadavers for ultrasound imaging. Imaging quality is appropriate while maintaining the characteristics of soft tissue suitable for practical training. It also has the advantage of lower formalin concentration than conventional fixatives, making it less harmful to users.

Decomposition of postmortem tissue usually makes it unusable for a long time and poses health risks to users [13,21]. The use of various chemicals as preservative materials to solve this problem affects the quality of radiographic images [30]. All the embalming solutions used in this study degraded the medical imaging quality. Image quality degradation is believed to be due to failure of the embalming process, in which the chemicals in the embalming solution may not inhibit tissue decomposition as the chemicals may not have penetrated the cadaver sufficiently, and the chemical components in the embalming solutions may have worsened the image quality [6].

In previous studies, the quality of radiographic images from embalmed cadavers was assessed without comparison with the quality of antemortem images [9,20]. However, the degradation in image quality can be misjudged due to the embalming solutions, even if it is due to the pathological condition of the subjects. Recently, several studies have compared cadaveric and antemortem images [6,31]. In addition, to assess whether long-term use was available for education in various subjects, image quality was compared over three months.

**Figure 7:**
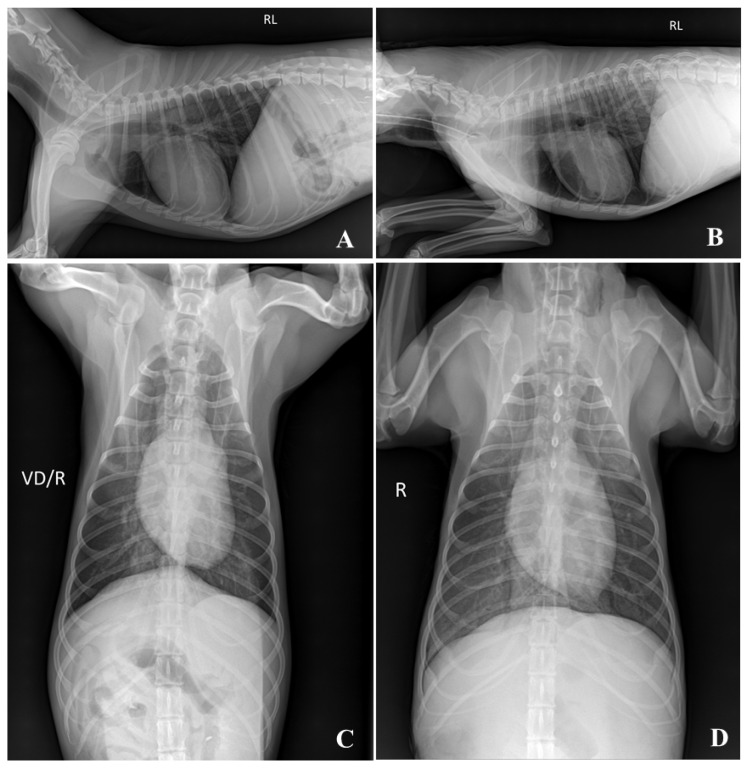
Thoracic radiographic images of the FS embalmed cadaver. Fixed as maximal inflated lung condition with intubation of the endotracheal tube. (**A**,**C**) = pre-imaging; (**B**,**D**) = 12 weeks. RL: Right lateral recumbency; VD/R: Ventodorsal recumbency/Right; R:Right. The mean score of imaging quality was 3.00 for Pre and 2.50 for 12 weeks.

Plain radiography is preferred because it can quickly determine the skeletal structure and retention of gases and liquids in the lungs and digestive organs. In plain radiographic images, all embalming solutions scored >1.3, although the image quality of the TS was the lowest. When intubation with an endotracheal tube was performed to assess whether the lungs had remained inflated, the thoracic cavity was maintained at maximal inflation (Figure 7). However, FS cadavers are difficult to position because of their hard skin and joint tissues [18,22]. The joint flexibility of the TS cadaver makes radiography easier [25]. Creating flexed and extended positions in axial and appendicular joint images is hypothesized to be useful for training.

Using an ultrasound, deterioration of image quality was observed for all embalming solutions. This is different from a previous study that showed improvements in the quality of ultrasound images from post-embalming cadavers by replacing the gas generated during decomposition with an infusion of preservative fluid [6,29]. This is explained by storage in a refrigerator at 2–4 °C instead of soaking cadavers in a bath with embalming solution or storing below freezing point in previous studies [25,30]. While the score for the SS group was 1.79 at 0 week, the visibility of the ultrasound image was very poor and scored less than one at 6 and 12 weeks due to gas production and decay.

The high concentration of formalin in FS reduces the flexibility of soft tissues such as the skin and joints [18,21]. There was a gas artifact at the interface between the ultrasonic probe and postmortem sample. In addition, it was difficult to apply pressure to the cadaver during ultrasound examination. These results indicate the limitations of using FS in the education of ultrasound and ultrasound-induced techniques required for veterinary training. Therefore, TS is considered the most suitable for practical ultrasonic induction training, which is consistent with the results of previous studies [1].

Regarding CT images, the scores indicated that the quality of the images deteriorated after embalming in all solutions. The lowest score was 2.42 for FS, 1.62 for TS, and 1.89 for SS. This means that CT images had the best quality after embalming among the three imaging modalities and were appropriate for training using cadavers. Similar to plain radiography, cadavers with TS maintain good joint mobility, making it possible to assume appropriate positions. However, it is difficult to identify vascular structures, such as the aorta and caudal vena cava, owing to the diameter depletion of blood vessels. Cadavers with TS and SS have limitations in observing pulmonary structures because of the fluid generation in the lung parenchyma. The thoracic cavity with maximal inflation was well maintained in the FS cadaver with endotracheal tube intubation, which is suitable for understanding pulmonary anatomy.

Although the present study involved the sacrifice of healthy dogs and received IACUC approval, the ethical implications of this practice remain a concern. Initially, the sacrifice of healthy animals was unavoidable, as obtaining anatomically normal specimens was critical for embalming and subsequent educational applications. As this research represents a continuation of our previous work, it complies with the reduction principle among the 3Rs [7]. In our study, we compared various embalming solutions and identified one that demonstrated high utility for veterinary imaging education using canine cadavers. Subsequently, these cadavers were employed to determine the optimal solution for veterinary anatomy practical education. The core of the current request is to explicitly declare that healthy animals will no longer be sacrificed for research purposes. To address ethical concerns in future research, we propose utilizing animals that have been euthanized due to illness or unavoidable circumstances, provided that their anatomical integrity is maintained. This approach ensures that normal animal morphology, which is critical for veterinary education, is preserved while mitigating ethical issues. Ultimately, based on our current findings regarding the effectiveness of a specific embalming solution, we anticipate that the sacrifice of healthy animals will no longer be necessary in future studies. 

The current study has several limitations. First, only a small number of cadavers were included. With strict 3R policies, sacrificing several animals for research is difficult. A small sample size impedes the aim of the study to observe statistically significant differences, providing limited estimates of variation in samples unless the differences are quite large. Therefore, careful interpretation is important when surveying statistical results, and additional multicenter studies should be conducted to reliably evaluate the findings of this study. Second, the cadavers were stored in a refrigerator without being immersed in the embalming solution. Thus, there is a possibility that decomposition may have occurred faster than that in previous studies [30]. Thirdly, this study was conducted over a relatively long interval between examinations. Further studies with short intervals are required to identify when the image quality is degraded and is no longer appropriate for training. 

## 5. Conclusions

To the best knowledge, this is the first study comparing images over time to assess the long-term usefulness of embalmed canine cadavers in education on veterinary medical imaging. FS provides excellent preservation of cadavers; however, its high concentration reduces the flexibility of soft tissues, such as skin and joints, and poses risks to human health. TS preserves soft tissue flexibility and facilitates cadaver positioning, offering superior utility for ultrasound imaging due to joint flexibility and lower formalin concentration, which reduces health risks. However, TS is limited in preserving vascular structures and pulmonary anatomy due to fluid accumulation in the lung parenchyma. Saturated salt solution is a cost-effective and simple alternative, but it showed rapid degradation of ultrasound image quality over time due to gas production and tissue decay. This makes it less suitable for long-term use in training. In conclusion, TS is the most appropriate embalming solution for all imaging techniques used in veterinary medical imaging education.

## Figures and Tables

**Table 1:** Composition of embalming solutions.

Formalin Solution		Saturated Salt Solution		Thiel’s Solution	
Solution Formula	Amount	Solution Formula	Amount	Solution Formula	Amount
36–40% Formalin	0.25 L	Sodium chloride	4 kg	1. Stem solution A	
Ethanol	1.5 L	36–40% Formalin	0.134 L	4-Chloro-3-methylphenol	26.4 g
Phenol	0.3 L	Isopropyl alcohol	0.8 L	Propylene glycol	0.264 L
Glycerol	0.1 L	Phenol	0.04 L	2. Stem solution B	
Water	3.05 L	Glycerol	0.1 L	Ammonium nitrate	1 kg
Total	5 L	Water	3.86 + 0.66 L	Hot water	1.6 L
		Total	5 L	3. Stem solution C	
				Boric acid	148 g
				Potassium nitrate	248 g
				Hot water	2 L
				4. Stock solution	
				Stem solution A	0.264 L
				Stem solution B	1.6 L
				Stem solution C	2 L
				Propylene glycol	1.48 L
				Hot water	1.32 L
				5. Final solution	
				Stock solution	6.664 L
				Sodium sulfate	320 g
				36–40% Formalin	0.16 L
				Morpholine	0.12 L
				Ethanol	0.52 L
				Total	7.544 L

The embalming solutions were prepared as described previously [14].

**Table 2:** Inter-observer agreements for scoring imaging quality.

Modality	ICC	Interval for 95% ICC	*p*-Value
Plain radiography	0.893	0.629–0.969	<0.001
CT	0.978	0.925–0.994	<0.001

ICC, intraclass correlation coefficient. Statistical significance was set at *p* < 0.05.

**Table 3:** Plain radiographic images: Mean visibility scores captured at four time points using three different embalming solutions.

Embalming Solution	Pre	0 w	6 w	12 w
**FS**	2.69 ± 0.12	2.25 ± 0.35 ^a^	1.97 ± 0.40 ^a^	1.77 ± 0.23 ^a^
**TS**	2.75 ± 0.25	1.90 ± 0.02 ^b^	1.59 ± 0.25 ^b^	1.34 ± 0.27 ^b^
**SS**	2.64 ± 0.19	2.11 ± 0.43	1.76 ± 0.30	1.51 ± 0.37

Scores are presented as mean ± standard deviation. FS, formalin solution; TS, Thiel’s solution; SS, saturated salt solution. Pre, before euthanasia; 0 w, days after embalming; 6 w, 6 weeks after embalming; 12 w, 12 weeks after embalming. ^a^ The minimal difference value by comparison with “Pre” score among three embalming solutions. ^b^ The maximal difference value by comparison with “Pre” score among three embalming solutions.

**Table 4:** Ultrasonographic images: mean visibility scores taken at four time points using three different embalming solutions.

Embalming Solution	Pre	0 w	6 w	12 w
**FS**	2.93 ± 0.12	2.15 ± 0.35 ^a^	1.44 ± 0.50 ^a^	1.33 ± 0.57 ^a^
**TS**	2.94 ± 0.04	2.07 ± 0.10	1.36 ± 0.15	1.26 ± 0.34
**SS**	2.90 ± 0.10	1.79 ± 0.52 ^b^	0.82 ± 0.33 ^b^	0.64 ± 0.38 ^b^

Scores are presented as mean ± standard deviation. FS, formalin solution; TS, Thiel’s solution; SS, saturated salt solution. Pre, before euthanasia; 0 w, days after embalming; 6 w, 6 weeks after embalming; 12 w, 12 weeks after embalming. ^a^ The minimal difference value by comparison with “Pre” score among three embalming solutions. ^b^ The maximal difference value by comparison with “Pre” score among three embalming solutions.

**Table 5:** CT images: mean visibility scores taken at four time points using three different embalming solutions.

Embalming Solution	Pre	0 w	6 w	12 w
**FS**	2.90 ± 0.02	2.60 ± 0.02 ^a^	2.47 ± 0.02 ^a^	2.42 ± 2.04 ^a^
**TS**	2.90 ± 0.02	1.95 ± 0.12 ^b^	1.70 ± 0.06 ^b^	1.62 ± 0.10 ^b^
**SS**	2.89 ± 0.02	2.24 ± 0.15	2.01 ± 0.19	1.89 ± 0.21

Scores are presented as mean ± standard deviation. FS, formalin solution; TS, Thiel’s solution; SS, saturated salt solution. Pre, before euthanasia; 0 w, days after embalming; 6 w, 6 weeks after embalming; 12 w, 12 weeks after embalming. ^a^ The minimal difference value by comparison with “Pre” score among three embalming solutions. ^b^ The maximal difference value by comparison with “Pre” score among three embalming solutions.

## Data Availability

The data supporting the conclusions of this article will be made available by the authors on reasonable request.
